# Supplementary material for: Shark movements between islands in the Revillagigedo Archipelago and connectivity to other islands in the Eastern Tropical Pacific
Source: PLoS One. 2026 Feb 18;21(2):e0341840. doi: 10.1371/journal.pone.0341840 (PMC12915964; doi:10.1371/journal.pone.0341840)
Supplement: S1 Table — San Benedicto (=SB), Socorro (=SO), Clarion (=CL), Roca Partida (RP), Clipperton = (CP), Cocos (=CO), Galapagos (=GA), and Malpelo (=MA). (DOCX) [file pone.0341840.s001.docx]

| **No.** | **Sex** | **TL** | **Tagging** | | **Monitoring** | | **Detections** | | | | | | | | |
| --- | --- | --- | --- | --- | --- | --- | --- | --- | --- | --- | --- | --- | --- | --- | --- |
|  |  |  | **Date** | **Island** | **Duration** | | **(N)** | | | | | | | |  |
|  | **(F,M)** | **(cm)** | **year-mon-day** |  | **(days)** | **(yr)** | **SB** | **SO** | **CL** | **RP** | **CP** | **CO** | **GA** | **MA** | **Total** |
| 1 | F | 196 | 2010-11-13 | San Benedicto | 1192 | 3.3 | 728 | 51 | 0 | 11 | 0 | 0 | 0 | 0 | 790 |
| 2 | F | 198 | 2010-11-15 | Socorro | 1093 | 3.0 | 0 | 1511 | 0 | 0 | 0 | 0 | 4 | 0 | 1515 |
| 3 | M | 198 | 2010-11-15 | Socorro | 1928 | 5.3 | 0 | 1273 | 0 | 0 | 0 | 0 | 0 | 0 | 1273 |
| 4 | Unk | 150 | 2010-11-20 | Roca Partida | 17 | 0.0 | 12 | 0 | 0 | 220 | 0 | 0 | 0 | 0 | 232 |
| 5 | F | 160 | 2010-11-20 | Roca Partida | 0 | 0.0 | 0 | 0 | 0 | 4 | 0 | 0 | 0 | 0 | 4 |
| 6 | F | 150 | 2010-11-20 | Roca Partida | 130 | 0.4 | 0 | 0 | 0 | 1352 | 0 | 0 | 0 | 0 | 1352 |
| 7 | F | 197 | 2010-11-21 | Roca Partida | 783 | 2.1 | 4 | 0 | 0 | 97 | 0 | 0 | 0 | 0 | 101 |
| 8 | F | 200 | 2010-11-21 | Roca Partida | 612 | 1.7 | 0 | 0 | 6 | 59 | 0 | 0 | 0 | 0 | 65 |
| 9 | F | 205 | 2010-11-21 | Roca Partida | 537 | 1.5 | 0 | 0 | 0 | 17 | 0 | 0 | 0 | 0 | 17 |
| 10 | F | 199 | 2013-04-11 | San Benedicto | 868 | 2.4 | 1532 | 0 | 0 | 0 | 0 | 0 | 0 | 0 | 1532 |
| 11 | F | 191 | 2013-05-28 | San Benedicto | 899 | 2.5 | 44351 | 0 | 0 | 0 | 0 | 0 | 0 | 0 | 44351 |
| 12 | F | 203 | 2014-05-22 | San Benedicto | 540 | 1.5 | 7445 | 0 | 0 | 0 | 0 | 0 | 0 | 0 | 7445 |
| 13 | F | 224 | 2014-05-22 | San Benedicto | 462 | 1.3 | 19911 | 5 | 0 | 0 | 0 | 0 | 0 | 0 | 19916 |
| 14 | F | 198 | 2014-05-25 | San Benedicto | 382 | 1.0 | 52 | 0 | 0 | 0 | 0 | 0 | 0 | 0 | 52 |
| 15 | F | 190 | 2014-10-04 | San Benedicto | 136 | 0.4 | 6402 | 0 | 0 | 0 | 0 | 0 | 0 | 0 | 6402 |
| 16 | F | 180 | 2014-10-04 | San Benedicto | 6 | 0.0 | 96 | 0 | 0 | 0 | 0 | 0 | 0 | 0 | 96 |
| 17 | F | 180 | 2014-10-04 | San Benedicto | 68 | 0.2 | 616 | 0 | 0 | 0 | 0 | 0 | 0 | 0 | 616 |
| 18 | F | 214 | 2014-11-17 | San Benedicto | 356 | 1.0 | 14222 | 0 | 0 | 0 | 0 | 0 | 0 | 0 | 14222 |
| 19 | F | 195 | 2014-11-21 | San Benedicto | 320 | 0.9 | 5467 | 0 | 0 | 0 | 0 | 0 | 0 | 0 | 5467 |
| 20 | F | 202 | 2014-11-21 | San Benedicto | 356 | 1.0 | 6162 | 0 | 0 | 0 | 0 | 0 | 0 | 0 | 6162 |
| 21 | F | 214 | 2014-11-25 | San Benedicto | 350 | 1.0 | 7573 | 0 | 0 | 0 | 0 | 0 | 0 | 0 | 7573 |
| 22 | F | 206 | 2011-01-23 | Cocos | 1067 | 2.9 | 0 | 0 | 0 | 0 | 0 | 1014 | 403 | 0 | 1417 |
| 23 | F | 226 | 2011-01-23 | Cocos | 1062 | 2.9 | 0 | 0 | 0 | 0 | 0 | 1003 | 42 | 0 | 1045 |
| 24 | F | 187 | 2010-03-05 | Wolf | 732 | 2.0 | 0 | 0 | 0 | 0 | 203 | 0 | 164 | 0 | 367 |
| 25 | F | 150 | 2010-03-08 | Wolf | 56 | 0.2 | 0 | 0 | 0 | 0 | 0 | 0 | 1830 | 0 | 1830 |
| 26 | F | 180 | 2012-02-15 | Wolf | 430 | 1.2 | 0 | 0 | 0 | 0 | 0 | 1 | 181 | 0 | 182 |
| 27 | F | 224 | 2012-02-19 | Darwin | 965 | 2.6 | 0 | 0 | 0 | 0 | 0 | 0 | 4800 | 0 | 4800 |
| 28 | F | 180 | 2014-01-17 | Wolf | 351 | 1.0 | 0 | 0 | 0 | 0 | 0 | 61 | 1242 | 0 | 1303 |
| 29 | F | 198 | 2014-01-17 | Darwin | 438 | 1.2 | 0 | 0 | 0 | 0 | 0 | 12 | 8666 | 0 | 8678 |
| 30 | F | 183 | 2014-01-17 | Wolf | 41 | 0.1 | 0 | 0 | 0 | 0 | 0 | 0 | 1733 | 0 | 1733 |
| 31 | F | 183 | 2014-01-18 | Wolf | 437 | 1.2 | 0 | 0 | 0 | 0 | 0 | 7 | 1605 | 0 | 1612 |
| 32 | F | 223 | 2014-01-18 | Wolf | 428 | 1.2 | 0 | 0 | 0 | 0 | 0 | 19 | 1027 | 0 | 1046 |
| 33 | F | 189 | 2014-01-18 | Wolf | 0 | 0.0 | 0 | 0 | 0 | 0 | 0 | 0 | 1 | 0 | 1 |
| 34 | F | 190 | 2014-01-19 | Darwin | 206 | 0.6 | 0 | 0 | 0 | 0 | 0 | 0 | 155 | 0 | 155 |
| 35 | F | 216 | 2014-01-19 | Darwin | 134 | 0.4 | 0 | 0 | 0 | 0 | 0 | 42 | 1516 | 0 | 1558 |
| 36 | F | 224 | 2014-01-19 | Darwin | 30 | 0.1 | 0 | 0 | 0 | 0 | 0 | 0 | 754 | 0 | 754 |
| 37 | M | 214 | 2012-02-15 | Wolf | 833 | 2.3 | 0 | 0 | 0 | 0 | 0 | 1412 | 7482 | 0 | 8894 |
