# Supplementary material for: Shark movements between islands in the Revillagigedo Archipelago and connectivity to other islands in the Eastern Tropical Pacific
Source: PLoS One. 2026 Feb 18;21(2):e0341840. doi: 10.1371/journal.pone.0341840 (PMC12915964; doi:10.1371/journal.pone.0341840)
Supplement: S2 Table — San Benedicto (=SB), Socorro (=SO), Clarion (=CL), Roca Partida (RP), Clipperton = (CP), Cocos (=CO), Galapagos (=GA), and Malpelo (=MA). (DOCX) [file pone.0341840.s002.docx]

| **No.** | **Sex** | **TL** | **Tagging** | | **Monitoring** | | **Detections** | | | | | | | | |
| --- | --- | --- | --- | --- | --- | --- | --- | --- | --- | --- | --- | --- | --- | --- | --- |
|  |  |  | **Date** | **Island** | **Duration** | | **(N)** | | | | | | | | |
|  | **(F,M)** | **(cm)** | **year-mon-day** |  | **(days)** | **(yr)** | **SB** | **SO** | **CL** | **RP** | **CP** | **CO** | **GA** | **MA** | **Total** |
| 1 | Unk | 175 | 2010-05-05 | Roca Partida | 450 | 1.2 | 0 | 0 | 0 | 3692 | 0 | 0 | 0 | 0 | 3692 |
| 2 | Unk | 200 | 2010-05-06 | Roca Partida | 15 | 0.0 | 0 | 0 | 0 | 1263 | 0 | 0 | 0 | 0 | 1263 |
| 3 | Unk | 200 | 2010-05-06 | Roca Partida | 414 | 1.1 | 0 | 0 | 0 | 4648 | 0 | 0 | 0 | 0 | 4648 |
| 4 | F | 180 | 2010-05-05 | Roca Partida | 245 | 0.7 | 0 | 0 | 0 | 51389 | 0 | 0 | 0 | 0 | 51389 |
| 5 | F | 210 | 2010-11-14 | San Benedicto | 1096 | 3.0 | 7241 | 289 | 0 | 0 | 0 | 0 | 0 | 0 | 7530 |
| 6 | F | 300 | 2010-11-22 | Roca Partida | 297 | 0.8 | 0 | 0 | 0 | 745 | 0 | 0 | 0 | 0 | 745 |
| 7 | Unk | 250 | 2010-11-22 | Roca Partida | 57 | 0.2 | 0 | 0 | 0 | 6736 | 0 | 0 | 0 | 0 | 6736 |
| 8 | F | 200 | 2011-04-19 | Roca Partida | 1020 | 2.8 | 1 | 0 | 0 | 433 | 0 | 0 | 0 | 0 | 434 |
| 9 | F | 180 | 2011-04-22 | San Benedicto | 10 | 0.0 | 1635 | 0 | 0 | 0 | 0 | 0 | 0 | 0 | 1635 |
| 10 | F | 250 | 2011-04-22 | San Benedicto | 68 | 0.2 | 440 | 17 | 0 | 0 | 0 | 0 | 0 | 0 | 457 |
| 11 | M | 250 | 2011-04-18 | Roca Partida | 903 | 2.5 | 10 | 1 | 0 | 51082 | 0 | 0 | 0 | 0 | 51093 |
| 12 | Unk | 200 | 2011-04-22 | San Benedicto | 493 | 1.4 | 5776 | 4962 | 0 | 0 | 0 | 0 | 0 | 0 | 10738 |
| 13 | M | 111 | 2011-12-12 | Socorro | 840 | 2.3 | 0 | 7385 | 0 | 0 | 0 | 0 | 0 | 0 | 7385 |
| 14 | F | 180 | 2011-12-13 | Roca Partida | 421 | 1.2 | 0 | 0 | 0 | 4835 | 0 | 0 | 0 | 0 | 4835 |
| 15 | F | 200 | 2011-12-14 | Roca Partida | 194 | 0.5 | 0 | 142 | 0 | 26256 | 0 | 0 | 0 | 0 | 26398 |
| 16 | Unk | 200 | 2011-12-13 | Roca Partida | 68 | 0.2 | 0 | 7 | 0 | 7200 | 0 | 0 | 0 | 0 | 7207 |
| 17 | Unk | 180 | 2012-04-20 | Roca Partida | 200 | 0.5 | 0 | 0 | 0 | 3764 | 0 | 0 | 0 | 0 | 3764 |
| 18 | F | 180 | 2016-10-27 | Socorro | 247 | 0.7 | 5227 | 0 | 0 | 0 | 334 | 0 | 303 | 0 | 5864 |
| 19 | F | Unk | 2010-06-30 | Cocos | 334 | 0.9 | 0 | 0 | 0 | 0 | 0 | 537 | 33 | 0 | 570 |
| 20 | F | 251 | 2011-01-23 | Cocos | 334 | 0.9 | 0 | 0 | 0 | 0 | 0 | 208 | 273 | 0 | 481 |
| 21 | F | 258 | 2011-01-25 | Cocos | 1306 | 3.6 | 0 | 0 | 0 | 0 | 0 | 7186 | 332 | 0 | 7518 |
| 22 | F | 300 | 2012-06-28 | Cocos | 696 | 1.9 | 0 | 0 | 0 | 0 | 0 | 2757 | 581 | 0 | 3338 |
| 23 | Unk | Unk | 2011-03-19 | Cocos | 334 | 0.9 | 0 | 0 | 0 | 0 | 0 | 135 | 314 | 0 | 449 |
| 24 | Unk | Unk | 2012-09-30 | Cocos | 304 | 0.8 | 0 | 0 | 0 | 0 | 0 | 42 | 114 | 0 | 156 |
| 25 | Unk | Unk | 2013-11-15 | Cocos | 228 | 0.6 | 0 | 0 | 0 | 0 | 0 | 174 |  | 0 | 174 |
| 26 | Unk | Unk | 2013-11-17 | Cocos | 334 | 0.9 | 0 | 0 | 0 | 0 | 0 | 36 | 1454 | 0 | 1490 |
| 27 | Unk | Unk | 2014-07-02 | Cocos | 631 | 1.7 | 0 | 0 | 0 | 0 | 0 | 2474 |  | 0 | 2474 |
| 28 | Unk | Unk | 2016-08-26 | Cocos | 543 | 1.5 | 0 | 0 | 0 | 0 | 0 | 0 | 0 | 0 | 0 |
| 29 | F | 200 | 2009-03-15 | Darwin | 769 | 2.1 | 0 | 0 | 0 | 0 | 0 | 0 | 137 | 0 | 137 |
| 30 | F | 200 | 2009-08-07 | Wolf | 559 | 1.5 | 0 | 0 | 0 | 0 | 0 |  | 108 | 0 | 108 |
| 31 | F | 140 | 2010-01-28 | Santa Cruz | 569 | 1.6 | 0 | 0 | 0 | 0 | 0 |  | 376 | 0 | 376 |
| 32 | F | 150 | 2010-01-28 | Santa Cruz | 226 | 0.6 | 0 | 0 | 0 | 0 | 0 |  | 47 | 0 | 47 |
| 33 | F | 250 | 2010-01-29 | Baltra | 181 | 0.5 | 0 | 0 | 0 | 0 | 0 |  | 8 | 0 | 8 |
| 34 | F | 200 | 2010-03-08 | Wolf | 1 | 0.0 | 0 | 0 | 0 | 0 | 0 |  | 4 | 0 | 4 |
| 35 | F | 180 | 2010-03-10 | Roca Redonda | 0 | 0.0 | 0 | 0 | 0 | 0 | 0 | 0 | 0 | 0 | 0 |
| 36 | F | 220 | 2010-03-13 | Santa Cruz | 260 | 0.7 | 0 | 0 | 0 | 0 | 0 |  | 9579 | 0 | 9579 |
| 37 | F | 170 | 2010-03-13 | Santa Cruz | 692 | 1.9 | 0 | 0 | 0 | 0 | 0 |  | 7264 | 0 | 7264 |
| 38 | F | 160 | 2010-03-14 | Santa Cruz | 521 | 1.4 | 0 | 0 | 0 | 0 | 0 |  | 221 | 0 | 221 |
| 39 | F | 160 | 2010-03-14 | Santa Cruz | 0 | 0.0 | 0 | 0 | 0 | 0 | 0 |  | 1 | 0 | 1 |
| 40 | F | 200 | 2012-02-17 | Wolf | 292 | 0.8 | 0 | 0 | 0 | 0 | 0 | 2100 | 13466 | 0 | 15566 |
| 41 | F | 194 | 2012-02-18 | Wolf | 762 | 2.1 | 0 | 0 | 0 | 0 | 0 | 1429 | 7922 | 0 | 9351 |
| 42 | M | 245 | 2009-08-06 | Darwin | 858 | 2.4 | 0 | 0 | 0 | 0 | 0 | 0 | 1291 | 0 | 1291 |
| 43 | M | 170 | 2010-03-14 | Santa Cruz | 120 | 0.3 | 0 | 0 | 0 | 0 | 0 | 0 | 2103 | 0 | 2103 |
| 44 | Unk | 150 | 2008-07-26 | Wolf | 5 | 0.0 | 0 | 0 | 0 | 0 | 0 | 0 | 141 | 0 | 141 |
| 47 | F | 230 | 2011-02-24 | Malpelo | 1 | 0.0 | 0 | 0 | 0 | 0 | 0 | 0 | 0 | 25 | 25 |
| 48 | F | 200 | 2011-02-24 | Malpelo | 147 | 0.4 | 0 | 0 | 0 | 0 | 0 | 429 | 0 | 2437 | 2866 |
| 49 | F | 227 | 2014-12-13 | Malpelo | 425 | 1.2 | 0 | 0 | 0 | 0 | 0 | 0 | 207 | 0 | 207 |
| 50 | M | 209 | 2014-12-13 | Malpelo | 3 | 0.0 | 0 | 0 | 0 | 0 | 0 | 0 | 5 | 0 | 5 |
| 51 | M | 210 | 2014-12-14 | Malpelo | 45 | 0.1 | 0 | 0 | 0 | 0 | 0 | 0 | 0 | 13 | 13 |
| 52 | Unk | Unk | 2008-03-08 | Malpelo | 474 | 1.3 | 0 | 0 | 0 | 0 | 0 | 0 | 0 | 0 | 0 |
