# Supplementary material for: Shark movements between islands in the Revillagigedo Archipelago and connectivity to other islands in the Eastern Tropical Pacific
Source: PLoS One. 2026 Feb 18;21(2):e0341840. doi: 10.1371/journal.pone.0341840 (PMC12915964; doi:10.1371/journal.pone.0341840)
Supplement: Supplementary file 3 [file pone.0341840.s003.docx]

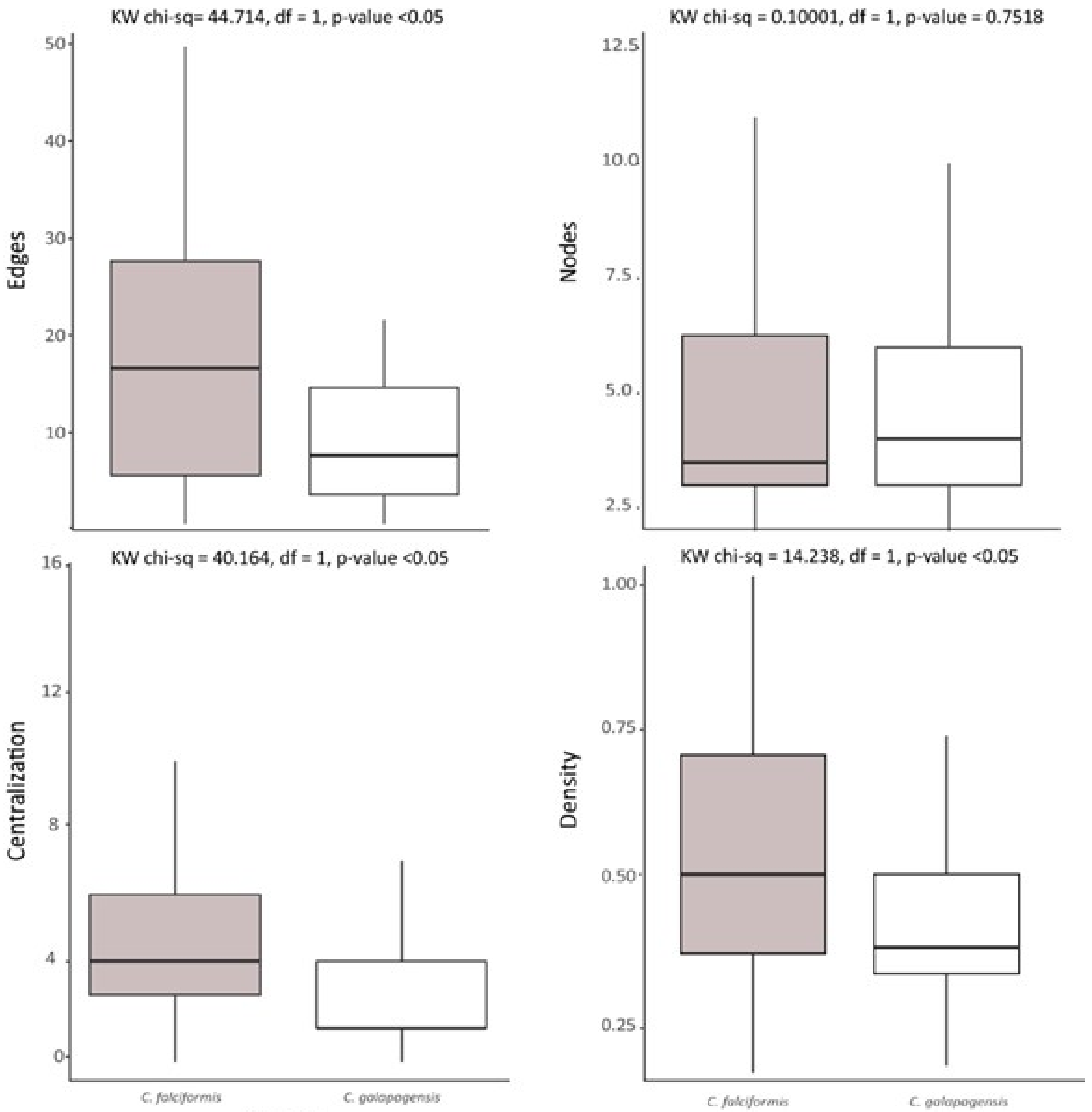

KW chi-sq= 44.714, df = 1, p-value <0.05
Edges
50
40
30
20
10
KW chi-sq = 0.10001, df = 1, p-value = 0.7518
Nodes
12.5
10.0
7.5
5.0
2.5
KW chi-sq = 40.164, df = 1, p-value <0.05
Centralization
16
12
8
4
0
C. falciformis
C. galapagensis
Species
KW chi-sq = 14.238, df = 1, p-value <0.05
Density
1.00
0.75
0.50
0.25
C. falciformis
C. galapagensis
Species
