# Supplementary material for: Shark movements between islands in the Revillagigedo Archipelago and connectivity to other islands in the Eastern Tropical Pacific
Source: PLoS One. 2026 Feb 18;21(2):e0341840. doi: 10.1371/journal.pone.0341840 (PMC12915964; doi:10.1371/journal.pone.0341840)
Supplement: Attachment — Submitted filename: Journal requirements and responses to the reviewers.docx [file pone.0341840.s005.docx]

| **Journal requirements** | **Author’s response** |
| --- | --- |
| Please submit your revised manuscript by Sep 06 2024 11:59PM. If you will need more time than this to complete your revisions, please reply to this message or contact the journal office at <a href="mailto:plosone@plos.org">plosone@plos.org. When you're ready to submit your revision, log on to https://www.editorialmanager.com/pone/ and select the 'Submissions Needing Revision' folder to locate your manuscript file.  Please include the following items when submitting your revised manuscript:   - A rebuttal letter that responds to each point raised by the academic editor and reviewer(s). You should upload this letter as a separate file labeled 'Response to Reviewers'. - A marked-up copy of your manuscript that highlights changes made to the original version. You should upload this as a separate file labeled 'Revised Manuscript with Track Changes'. - An unmarked version of your revised paper without tracked changes. You should upload this as a separate file labeled 'Manuscript'.   If you would like to make changes to your financial disclosure, please include your updated statement in your cover letter. Guidelines for resubmitting your figure files are available below the reviewer comments at the end of this letter. | The manuscript has been rewritten considering all the comments.  We include the rebuttal letter and marked up copy with tracked changes and an unmarked version. |
| 1. When submitting your revision, we need you to address these additional requirements.  Please ensure that your manuscript meets PLOS ONE's style requirements, including those for file naming. The PLOS ONE style templates can be found at  https://journals.plos.org/plosone/s/file?id=wjVg/PLOSOne_formatting_sample_main_body.pdf and  https://journals.plos.org/plosone/s/file?id=ba62/PLOSOne_formatting_sample_title_authors_affiliations.pdf. | The style requirements for file naming have been considered. |
| 2. Thank you for stating the following financial disclosure:   Please state what role the funders took in the study. If the funders had no role, please state: "The funders had no role in study design, data collection and analysis, decision to publish, or preparation of the manuscript." If this statement is not correct you must amend it as needed.  Please include this amended Role of Funder statement in your cover letter; we will change the online submission form on your behalf. | The financial disclosure has changed, specifying the role of the funders. |
| 3. Thank you for stating the following in the Acknowledgments Section of your manuscript:   Please remove any funding-related text from the manuscript and let us know how you would like to update your Funding Statement. Currently, your Funding Statement reads as follows:   [The authors (MH and JK) would like to thank the International Community Foundation, Ocearch, Chris Fischer Productions and National Geographic for providing the initial funding to tag many sharks and set up the acoustic receiver array in the Revillagigedo Archipelago. We are gratefiul to Club Cantamar, Fins Attached (Alex Antoniou), Sharks Mission France and Quino El Guardian for supporting our expeditions to the islands. We also thank the Alliances WWF-Telmex-Telcel and WWF-Fundación Carlos Slim and Ocean Blue Tree for additional support for this project].  Please include your amended statements within your cover letter; we will change the online submission form on your behalf. | The Acknowledgments section has been changed, removing the funding related text. |
| 4. Please provide a complete Data Availability Statement in the submission form, ensuring you include all necessary access information or a reason for why you are unable to make your data freely accessible. If your research concerns only data provided within your submission, please write "All data are in the manuscript and/or supporting information files" as your Data Availability Statement. | We completed the Data Availability Statement in the submission form. |
| 5. Please amend the manuscript submission data (via Edit Submission) to include author A. Peter Klimley and Felipe Galván-Magaña. | We amended the manuscript submission data to include two authors. |
| 6. Your ethics statement should only appear in the Methods section of your manuscript. If your ethics statement is written in any section besides the Methods, please move it to the Methods section and delete it from any other section. Please ensure that your ethics statement is included in your manuscript, as the ethics statement entered into the online submission form will not be published alongside your manuscript. | The ethics statement has been moved to the Methods section and deleted from any other section. |
| 7. We notice that your supplementary figures are uploaded with the file type 'Figure'. Please amend the file type to 'Supporting Information'. Please ensure that each Supporting Information file has a legend listed in the manuscript after the references list. | The supplementary figure amended the file type to 'Supporting Information'. |
| 8. Please include captions for your Supporting Information files at the end of your manuscript, and update any in-text citations to match accordingly. Please see our Supporting Information guidelines for more information: http://journals.plos.org/plosone/s/supporting-information. | We included captions for your Supporting Information files at the end of your manuscript. |

| Reviewers' comments: Reviewer #1 | Author’s response |
| --- | --- |
| This manuscript investigated the degree of movement between islands in Marine Protected Areas (MPAs) of eastern tropical Pacific using long period (2010-2018) of ultrasonic telemetry dataset for 82 individuals of two pelagic sharks (Galapagos sharks and Silky sharks). I think that the understanding of the horizontal movement of pelagic sharks is important to determine appropriately the size, number and place of the MPAs. The authors attempted to summarize the movement patterns of type 1, 2, and 3 using the network analysis. Probably, I think that the figs.5 and 6 indicate the main results of your study. I understood that the network among research points was well constructed for each species surrounding the MPAs, but it is difficult to evaluate the effectiveness of the MPAs from your analysis because the ultrasonic telemetry can only detect the movement around the location of the receptors which are placed only near the islands, however, both species are highly migratory species, so that it is uncertain whether it is enough to protect the shark species using the MPAs based on the results of this study. | The comments of the reviewer 1 have been considered in the last version of the manuscript. |
| For introduction, I think the authors should mention the reasons, the pelagic sharks stay in the similar sites or move to the different islands, from the perspective of the biology and ecology using the information about the past studies, otherwise it is unclear the effectiveness of the setting of the MPA in this region. | In the introduction, we now mentioned the reasons why pelagic sharks stay in similar sites or move to different islands (Line 165). |
| Overall, the quality of the figures is low, and the resolution of figures is low as well. Therefore, it is difficult to understand (e.g., Figs1-3,7). Furthermore, there is no figure captions for all figures. | The figures have been uploaded in a higher resolution. |
| L70-71: I don’t think that the MPAs are always more effective management strategies for migratory species than any other management measures such as TAC and TAE. Please mention the reason. | In the particular case of the ETP, we mentioned the reasons for MPAs are effective management strategies for migratory species supported win addition to other measures such as TAC and TAE. |
| L85: Add “Figure 1” to the end of sentence. For figure1: Please insert the latitude and longitude for the maps. | Added. |
| L98-109: What is the main reason to conduct the inter-island movement in the ETP for scalloped hammerhead sharks? Please clarify. | We clarified it in the paragraph. |
| L112-114: Do you want to mention about the sharks or general marine species? Please clarify. | We clarified it in the paragraph. |
| L120: What is the difference between “the eastern Pacific” and “ETP”? | None. We standardized the term. |
| L123: ecology -> behavioral ecology | Changed. |
| L124-125: What is the network analysis? Is the movement frequency enough to identify the important movement paths? | A better definition of network analysis is included. We explained why the movement frequency is a good indication to identify important movement paths. |
| L150-151: This sentence means that this species is a coastal shark, not pelagic shark. | Modified. |
| L156-158: Add “Figure 1” to the end of sentence. | Added. |
| L162-163: There is no CP in the Figure 1. | Added. |
| L187: Why you didn’t do in the CA. | Added. |
| L215-216: Add “Figure 2” to the end of sentence. | Added. |
| L239: network analysis -> NA | Changed. |
| L251: What is the y axis in Fig.2 and I cannot identify each location from this figure. | Edited. |
| L253-281: To show the horizontal movement of each shark, the author should make the map including the pathway of each shark for the period tagged. | Included. |
| L283-295: It is difficult to understand the description of this paragraph from the Figs 5 and 6. | Edited. |
| L310-314: The authors should show the table on the values of eigenvalue. | Included. |
| L319: Which months are the wet months? | Included. |
| L321-322: The author should examine the effect of SST on the horizontal movement of both species. | Included. |
| L329-331: either of daytime -> night time | Included. |
| L345-350: I agreed that author should examine the site-fidelity of each species by different sex and life stages. | Considered. |
| L371-372: The authors discussed the characteristics of this individuals from the sex and life stages based on the total length. | Included. |
| L394-397: I think this is the shortcoming of this tools. | Changed. |
| L435: I cannot see the figure well. | A higher resolution figure is included. |
| L449: MPAS -> MPAs | Changed. |
| L457-462: The author should discuss the stock status based on the indicator analysis of silky sharks and the annual catches conducted by IATTC. | The discussion is included. |
| L468-470: Scientifically, the methodology used in this analysis cannot draw this conclusion. The author should use the environmental data in relation to the data of movements. | The conclusion has been changed according to the methods. |
| L472-276: It is true that this study showed that both species had a long-distance movement, but the authors didn’t show scientifically the clear reason for protecting the species using the MPA because there is no information about, what the size of the MPA (How many numbers of MPA; What connectivity etc.) is useful to protect for these species. | This statement is discussed in more detail in the manuscript. |

| **Reviewer #2:** | **Author’s response** |
| --- | --- |
| Review of “Shark movements between islands in the Revillagigedo Archipelago and the connectivity to other islands in the Eastern Tropical Pacific” | The reviewer comments have been considered in the latest version of the manuscript. |
| The paper has potentially important information on the movement and connectivity of widely spaced protected areas, which would have relevance for the difficult issue of how to protect long-ranging endangered animals visiting areas in between that are not protected. There seems to have been a large number of two species of sharks tagged, which should give a good overview of use of the areas. There were some problems with understanding the information in the manuscript. This began with the lack of captions for the figures and the apparent mis-labelling of the tables, making understanding the manuscript – not the authors’ fault. The figure captions was resolved by the journal, but it lead to further confusion with two versions of Figure 2 (below). As a result, the interpretation of the data became difficult to understand, not allowing any further review as the Discussion would not then make sense. I hope that the comments will help to make the paper stronger and one that can be used as a good reference. | We try to clarify the problems with understanding the information in the manuscript. We include the captions for the figures and the tables. |
| There seems to be a high degree of self-referencing (36%), which then does not allow the authors to support their arguments with a wider range of studies. The manuscript would be greatly enhanced and made stronger by discussing their work in a broader context of similar and relevant studies by their peers. | More references are included to make a stronger and wider range of studies. |
| The grammar in the Material and Methods section should be to be reviewed for clarity in understanding the text. Captions are generally meant to allow the reader to understand a table and figure without having to read the manuscript. The captions in this manuscript could use move information in them. | The section Material and Methods has been edited. |
| Line 162 discussed Cabo Pulmo National Park at length, but it is not mentioned in the five MPAs (Line 179) – is it meant to be a part of the study (Line 179-181)? | It was not included in the study because the goal was to describe the movements in insular ecosystems, however we consider interesting to show this detection in the Gulf of California. |
| Line 188: Is Table S1 available or is this Table 1? | The table is now included in the Supporting information. |
| Line 195 notes that maturity was reported for the sharks. This is not mentioned again in the manuscript, which would be very interesting in terms of areas each stage used and travel profile. The data on maturity is in Table S1 – but this does not seem to be available to read. | The maturity and life stage is now included and discussed in detail. |
| Line 250 notes that there are 37 silky and 50 Galapagos sharks, but Table 1 records 39 silky and 53 Galapagos sharks. | The number has been changed. The sharks that were not detected are not included. |
| Figure 2: as mentioned above, there were two different Figure 2s (cannot attach them), making interpretation of the data difficult. The Individual number on the figure and the number in the text do not seem to match on either figure. For example: Silky shark #14 tagged during 2013 (2014) at San Benedicto was not detected at Roca Partida or Socorro and not returning to San Benedicto, #15 was though. Silky #17 tagged at San Benedicto seemed to move to Socorro rather than Roca Partida. Without being clear on which figure is correct, it is difficult to understand the mismatch with the text in the Results – Movements section, or to go any further with the review until this is resolved. | The figures have been uploaded in a higher resolution and the numbers match in the figures. |
