# Supplementary material for: Shark movements between islands in the Revillagigedo Archipelago and connectivity to other islands in the Eastern Tropical Pacific
Source: PLoS One. 2026 Feb 18;21(2):e0341840. doi: 10.1371/journal.pone.0341840 (PMC12915964; doi:10.1371/journal.pone.0341840)
Supplement: Attachment — Submitted filename: Reply to comments_PlosOne.docx [file pone.0341840.s006.docx]

**Response to Reviewers**

We appreciate the time and effort from the reviewer for their careful reading of our manuscript and for the valuable feedback provided. The thoughtful suggestions have been discussed and considered, which have allowed us to improve the clarity and robustness of our work.

| **Comments** | **Reply to comments** |
| --- | --- |
| Grammar | We have carefuly improved the grammar with the support of native speaking English coauthors. |
| Reduce autocitations | We have eliminated a number of autocitations, however we had to keep several of these because most shark studies on movement and migratory patterns in the Eastern Tropical Pacific have been carried out by our research groups, namely MigraMar and Pelagios |
