# Supplementary material for: Shark movements between islands in the Revillagigedo Archipelago and connectivity to other islands in the Eastern Tropical Pacific
Source: PLoS One. 2026 Feb 18;21(2):e0341840. doi: 10.1371/journal.pone.0341840 (PMC12915964; doi:10.1371/journal.pone.0341840)
Supplement: Attachment — Submitted filename: Response to Reviewers_amended.docx [file pone.0341840.s007.docx]

Response to Reviewers

Manuscript ID: PONE-D-24-20949R2

| **Reviewer´s comments** | **Responses** |
| --- | --- |
| The MS has been storngly improved but authors should strongly reduce the self citations (they are more than the 31 %) before that it can be suitable for the publication. | Done. We have thoroughly addressed the primary concern by significantly reducing the number of self-citations throughout the manuscript.  We conducted a full-text review of the manuscript to identify all self-citations (references to works where Dr. Ketchum or any co-author was a contributing author). For each self-citation, we assessed its necessity. In many cases, we found suitable replacement citations from other research groups that supported the same point, often with more foundational or widely recognized studies. We replaced redundant self-citations with references to key papers from other authors that established the methodological or conceptual groundwork. In some instances, we were able to consolidate multiple points into a single, more appropriate citation. As a result of these edits, the proportion of self-citations has been reduced to well below the 31% threshold. |
| If the reviewer comments include a recommendation to cite specific previously published works, please review and evaluate these publications to determine whether they are relevant and should be cited. There is no requirement to cite these works unless the editor has indicated otherwise. | Thanks for the comment. Our goal was not only to meet the journal's criteria but to strengthen the manuscript by incorporating a broader range of foundational and contemporary literature. |
| Please review your reference list to ensure that it is complete and correct. If you have cited papers that have been retracted, please include the rationale for doing so in the manuscript text, or remove these references and replace them with relevant current references. Any changes to the reference list should be mentioned in the rebuttal letter that accompanies your revised manuscript. If you need to cite a retracted article, indicate the article’s retracted status in the References list and also include a citation and full reference for the retraction notice. | Done. The reference list now reflects a more balanced and robust integration of the wider scientific literature. All changes have been made in the 'Revised Manuscript with Track Changes' file, where the removed self-citations and their replacements are clearly highlighted. |
